# An insertion mutation in ABCB4 is associated with gallbladder mucocele formation in dogs

**DOI:** 10.1186/1476-5926-9-6

**Published:** 2010-07-03

**Authors:** Katrina L Mealey, Jonathan D Minch, Stephen N White, Kevin R Snekvik, John S Mattoon

**Affiliations:** 1 Department of Veterinary Clinical Sciences, College of Veterinary Medicine, Washington State University, Pullman, WA 99164-6610, USA; 2 USDA-ARS Animal Disease Research Unit, Pullman, WA 99164-6630, USA; 3 Department of Veterinary Microbiology & Pathology, College of Veterinary Medicine, Washington State University, Pullman, WA 99164, USA; 4 Center for Integrated Biotechnology, Washington State University, Pullman, WA, 99164, USA

## Abstract

**Background:**

ABCB4 functions as a phosphatidylcholine translocater, flipping phosphatidylcholine across hepatocyte canalicular membranes into biliary canaliculi. In people, ABCB4 gene mutations are associated with several disease syndromes including intrahepatic cholestasis of pregnancy, progressive familial intrahepatic cholestasis (type 3), primary biliary cirrhosis, and cholelithiasis. Hepatobiliary disease, specifically gallbladder mucocele formation, has been recognized with increased frequency in dogs during the past decade. Because Shetland Sheepdogs are considered to be predisposed to gallbladder mucoceles, we initially investigated *ABCB4 *as a candidate gene for gallbladder mucocele formation in that breed, but included affected dogs of other breeds as well.

**Results:**

An insertion (G) mutation in exon 12 of canine *ABCB4 *(*ABCB*4 1583_1584G) was found to be significantly associated with hepatobiliary disease in Shetland Sheepdogs specifically (P < 0.0001) as well as other breeds (P < 0.0006). *ABCB*4 1583_1584G results in a frame shift generating four stop codons that prematurely terminate ABCB4 protein synthesis within exon 12, abolishing over half of the protein including critical ATP and a putative substrate binding site.

**Conclusions:**

The finding of a significant association of *ABCB*4 1583_1584G with gallbladder mucoceles in dogs suggests that this phospholipid flippase may play a role in the pathophysiology of this disorder. Affected dogs may provide a useful model for identifying novel treatment strategies for ABCB4-associated hepatobiliary disease in people.

## Background

Bile is produced by the collective actions of a number of transporters located on the canalicular membrane of hepatocytes [1]. Active transport of biliary solutes creates an osmotic force that attracts water through tight junctions and aquaporins in the hepatocyte membrane [2,3]. Bile salts are the most important biliary solute. Other important solutes of bile include cholesterol and phospholipids. The presence of phospholipids, phosphatidylcholine (PC) in particular, in the biliary lumen is crucial for protecting the epithelial cell membranes lining the biliary system from the cytotoxic detergent actions of bile salts [3-5]. Bile salt cytotoxicity is substantially reduced in the presence of PC owing to the formation of mixed micelles (PC + bile salts) rather than simple micelles (bile salts only). Thus, a decrease in the amount of biliary PC leads to injury of epithelial cells lining the biliary system [6].

ABCB4 functions exclusively as a phospholipid translocator [6]. ABCB4 is expressed on cannalicular membranes of hepatocytes where it translocates PC from the hepatocyte to the biliary canalicular lumen [7]. Proper function of ABCB4 is critical for maintaining hepatobiliary homeostasis as evidenced by the myriad of diseases that occur when polymorphisms of ABCB4 cause complete or partial protein dysfunction. ABCB4 deficiency is associated with a variety of hepatobiliary disorders in people including progressive familial intrahepatic cholestasis (PFIC type 3), cholelithiasis, and cholestasis of pregnancy [4,8-10]. *Abcb*4-/- mice, in which *Abcb*4 function is lacking entirely, also develop severe hepatobiliary disease that starts at a few weeks of age and progresses throughout life [11,12].

Hepatobiliary disease in dogs has been recognized with increased frequency during the past several years. In particular, gallbladder mucoceles (mucinous hyperplasia or mucinous cholecystitis) have been documented to be an increasingly important cause of hepatobiliary disease in dogs [13-15]. Histopathologic findings associated with ABCB4 associated diseases in people, including intrahepatic cholestasis, cholecystitis, and periportal inflammation [13,16,17], are not commonly reported in dogs with gall bladder mucoceles. Additionally, gallbladder mucoceles are not a component of ABCB4 linked syndromes in people or mice. Gallbladder mucoceles, which occur rarely in people, are often associated with extrahepatic bile duct obstruction. The etiology of gallbladder mucoceles in dogs has not yet been identified, but extrehepatic bile duct obstruction is not commonly associated with this disorder [14,15]. Gallbladder mucoceles may result from chronic injury to the epithelial lining of the biliary system since hypersecretion of mucin is the typical physiologic response of any epithelial lining to injury.

Recently Shetland Sheepdogs were identified as a breed that is predisposed to gallbladder mucocele formation, suggesting a genetic predisposition [13]. Because ABCB4 dysfunction is associated with hepatobiliary disease in people and mice, we postulated that a defect in canine *ABCB*4 might be responsible for gallbladder mucocele disease in dogs, and Shetland Sheepdogs in particular. Therefore, we sequenced canine *ABCB*4 in affected and unaffected Shetland Sheepdogs as well as affected and unaffected dogs of other breeds.

## Methods

### Collection of DNA from affected and unaffected individuals

All work was approved by the institutional Animal Care and Use Committee. Collection of DNA from affected Shetland Sheepdogs was accomplished by soliciting owners' cooperation. In order to cast a wide net, owners of dogs with confirmed (ultrasound, surgery, or histopathology) or suspected (elevated liver enzymes - alkaline phosphatase, alanine aminotransferase and/or gamma glutamyl transferase -, total bilirubin, cholesterol and/or triglycerides) gallbladder disease were asked to submit a cheek swab, copy of the dog's pedigree, and copy of the dog's medical record. Contact of Shetland Sheepdog owners was made through the American Shetland Sheepdog Association. For collection of unaffected Shetland Sheepdogs, an additional request for DNA from healthy Shetland Sheepdogs (with confirmatory medical records) was made. For collection of DNA from affected dogs of any breed, records from the Washington Animal Disease Diagnostic Laboratory were searched for canine patients with histopathologic confirmation of gallbladder mucocele. For collection of DNA from unaffected dogs of any breed, a specific solicitation through the Washington State University College of Veterinary Medicine was made for healthy dogs (no history of gallbladder disease) over 9 years of age. In order to increase our confidence in designating a dog as "unaffected", we recruited dogs (Shetland Sheepdogs and other breeds) greater than 9 years of age. While this may have limited the number of dogs included in the study, it more accurately reflected a dog's true phenotype (affected *vs*. unaffected). A dog was considered 'affected' if a gallbladder mucocele was diagnosed using previously established criteria[13], which included at least one of the following (in order of increasing stringency); ultrasound report by a boarded veterinary radiologist (n = 3), surgical report (n = 5), or histopathologic report (n = 7). Dogs with no evidence of gallbladder disease as determined by a normal serum chemistry panel and no apparent physical examination abnormalities were considered 'unaffected'.

### Sequencing of canine *ABCB*4

Exons 1 through 26 of canine *ABCB*4 were sequenced after PCR amplification of genomic DNA from affected and unaffected Shetland Sheepdogs. Table 1 contains the sequences of the oligonucleotide primers. Purified PCR amplicons were sequenced with an Applied Biosystems ABI 3730 sequencer (Foster City, CA). Affected and unaffected dogs of other breeds (non-Shetland Sheepdogs) were sequenced only at exon 12. DNA from all dogs except the 3 affected non-Shetland Sheepdogs was extracted from cheek swab samples. Formalin-fixed, paraffin embedded liver tissue was used for extraction of DNA from these 3 dogs. Samples were processed first using the RiboPure RNA extraction kit (Ambion, Foster City, CA) until step C3. The interphase from this step (containing DNA and protein) was then subjected to DNA extraction using the DNeasy Blood and Tissue Kit (Qiagen, Alameda, CA).

**Table 1:** Primers used for amplifying canine ABCB4.

Exon	Forward Primer	Reverse Primer	Product Size
1	TTC AGT TGG CTA TGA AAC ATT TGG	AGA CTA TCT TAA AGC ACT GAC TCC	165
2	CCA AAA AAC ATA TAG TTT TGG GGA	GTC ATC TAG AAG TGC AAA CCA TTA AAC	302
3	CCT AGT AAC ACC TAT TAA TAG TTC AGC C	CTC TGT AAG TTT GCA ATT ATT CTC	202
4	CTT CCT GAA AGA GAT GAA TAA AGA AC	CAA AAG TAT GAC ATA AAT GAT ACA CTT AC	225
5	GAA GAC CTC CTG CCT GTA ACC ACT	CAC ATG TGA AAA TGT TCC CGT TTC	201
6	CAT GAA TGT TTC TTC TCT GTC CAG	GGT TCT TTG AAC CAG TGG AC	143
7	GGC TAT GAT TAT GGA CTG TTT TCT TG	GGT TTC TTC ACG AAT ATT AGA AAG AC	208
8	GCT TAT AAC TTC TTC TTG TGT TCT TTT G	GTG CAA GCC TCA AGG AAT TTT TTT TG	143
9	CCT TAA AAG TGC AGT TGG TTG	GAA ATA AAA CCT GCC ACA GG	249
10	CGT GAA GAG TGT TCT CTT TCT CTC	GCA GGG CTA ATT GGT AGC	177
11	CTT GAT GCT TTA GAT GTC AGA TGG	CTC ACT TGC CTG AAG TCA AAG	278
12	GAG ATA CAT CAG GAG CTC CTC C	CAG GTG TTT CGG GTT GAC TG	189
13	GTA ACC CTG TTG CAT CAC AC	CTC AGC ATG GCA TTA GCT GC	239
14	CAA CTT AAC ATT TTC TCT TCT TTC AG	GGA ATC ACT TGT GCC TGC	256
15	CCA CTT TCT CCT GAT TCT CCT G	GGT GAA GCT GGC ATG AGA AC	219
16	CTC TCT CTG GCT CTC ATG	CTC TAA TAG AAT GTG GAC TCG AG	188
17	CTG ATG ATC AAA AGG GAC AAT C	GGA CTT CTC AAG TGC ACA C	118
18	GAA GGT GTG TTT TGT GCC ACA G	CCC TTT CTG TCT CTC AAA TGG G	141
19	CAT GGC TCC CTC TTT GCT TTT GC	CTC ACT GAA GCC TTC TTT GAC CCA C	212
20	CGT TAT CCA GAA GTA AAA GCC C	CCT CAG GAA AGT ACT AGG GTC	159
21	CCA GTC AAC TAC ACT AGA AGC TG	GAA CAA GTG AGT TTT TTC CAC CC	260
22	GGT AAG CAC TAT GTC TTT GGA C	CAT TCA CCA GAC AGC AGA GAA C	222
23	CAG ACC AAT TAT AAT AGC AAC ATT AAC	GCC TTA AAT AAG GTA CTA ACT TAA GC	227
24	GAT ACC CAC ATG TCA CAA TGT TCC	TCC TGG TGC CAC TAC ATA GAC	402
25	GTC CTA TAC CAA GTC ATG AGG AC	GGA AAC AGA GTG GAA AGA CC	179
26	GGA ACT AAC TGT AGA CTA TAA TGC	GCT ATC TTA TCA ACA CCA AAT GG	393

### Allele specific PCR

In order to confirm the insertion mutation in exon 12 (*ABCB*4 1583_1584G), allele specific primers were designed (mutant: forward 5'- CCTGGTTCGCAACCCTAAGATCCG, reverse 5'- GCAATGTGGCCTGACAGAAAGGGGAAATC; wildtype: forward 5'- CCTGGTTCGCAACCCTAAGATCC, reverse 5'- GCAATGTGGCCTGACAGAAAGGGGAAATC) to amplify a 202 bp amplicon. This also allowed confirmation of individual genotype.

### Statistics

Association of genotype and gallbladder mucocele status was analyzed using the frequency procedure of SAS 9.2 (SAS Institute, Cary, NC), specifying Fisher's exact test and exact confidence intervals for the odds ratio.

## Results

### Collection of affected and unaffected individuals

**Figure 1:**
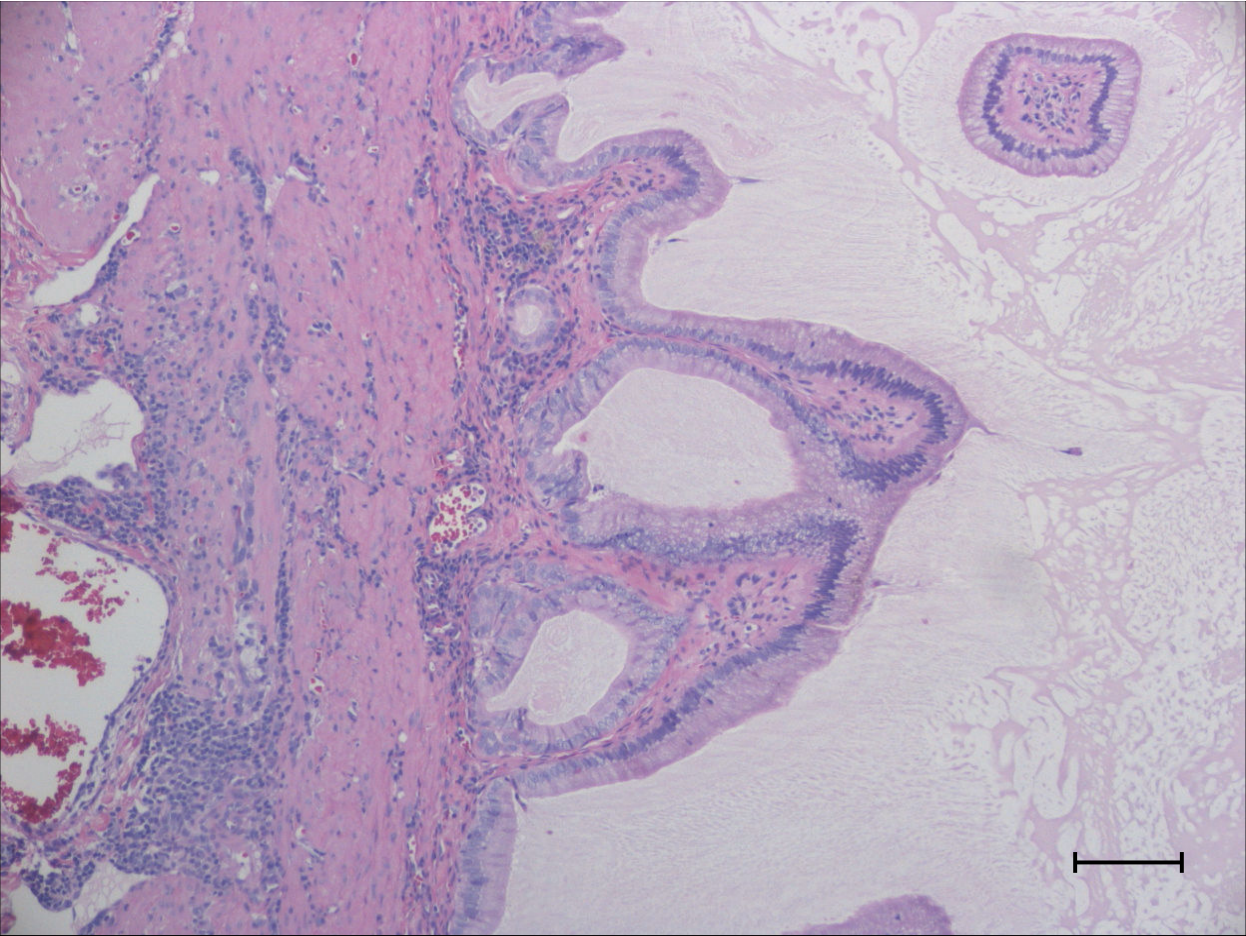
**Gall Bladder**. There is distention of the gall bladder with abundant luminal accumulations of mucus interspersed with scant amounts of bile. The mucosa of the gall bladder is lined by moderately hyperplastic columnar epithelial cells with accentuation of the normal folds by accumulations of mucus. Within the lamina propria and the tunica muscularis there are occasional multifocal to perivascular accumulations of lymphocytes and rare plasma cells. Hematoxylin and eosin staining. Bar = 250 μm.

Samples from 15 affected and 21 unaffected Shetland Sheepdogs were sequenced. Diagnosis of gallbladder mucocele was confirmed by ultrasound in 3 dogs, by surgery in 5 dogs, and by histopathology in 7 dogs (Figure 1). Median age of Shetland Sheepdogs with a diagnosis of gallbladder mucocele was 9 years (range 5-12), which is similar to previous reports [13,15]. Ages for all the 21 unaffected Shetland Sheepdogs were not available, but the median age for those dogs whose ages were known (n = 12) was 9.5 years of age (range 5-14). Ages and breeds of the 3 affected non-Shetland Sheedogs are as follows: Cairn Terrier (11 years), Cocker Spaniel (13 years) and Pomeranian (11 years). Ages and breeds of the 20 unaffected non-Shetland Sheepdogs are indicated in Table 2.

**Table 2:** Breed and age of unaffected dogs (non Shetland Sheepdogs).

Breed	Number of Dogs	Age(years)
Afghan Hound	3	9.5; 10; 10
Asluki	1	12
Australian Shepherd	1	10
Brittany Spaniel	1	11
Corgi	1	9
English Cocker Spaniel	1	12
Golden Retriever	1	9.5
Jack Russell Terrier	1	9
Kelpie	1	13
Labrador Retriever	3	9; 9.5; 9.5
Miniature Pinscher	2	10; 13.5
Mixed Breed	1	10
Pitt Bull	1	15
Shih Tzu	1	14
Standard Poodle	1	10

### Sequencing of Canine *ABCB*4

**Figure 2:**
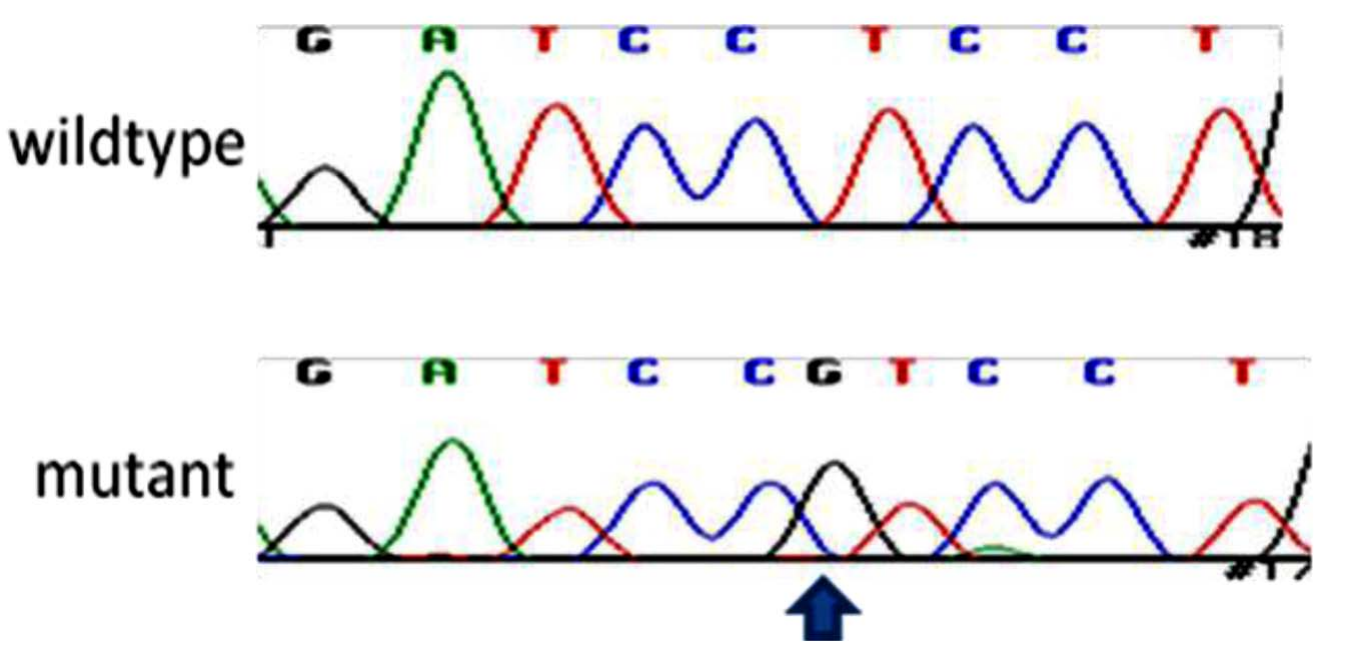
**Electropherograms for wildtype and mutant canine *ABCB*4**. The insertion is indicated by an arrow.

Sequencing of all exons (1 to 26) of canine *ABCB4 *was performed on genomic DNA from cheek swab samples (Shetland Sheepdogs) or from archived liver tissue (affected dogs that were not Shetland Sheepdogs). A single base pair insertion (G) was identified in exon 12 (Figure 2) in 14 of 15 affected Shetland Sheepdogs, 1 of 21 unaffected Shetland Sheepdogs, and 3 affected dogs of other breeds (Cairn Terrier, Cocker Spaniel, and Pomeranian). The insertion mutation (*ABCB*4 1583_1584G) is significantly associated (P < 0.0001) with the diagnosis of gallbladder mucocele in Shetland Sheepdogs, with an odds ratio of 280 (95% CI 12.7-12,350). In other dog breeds, *ABCB*4 1583_1584G is also significantly associated with the diagnosis of gallbladder mucocele (P < 0.0006). The frame shift generated by the insertion results in 4 premature stop codons within exon 12. The full canine *ABCB*4 gene contains 26 exons which encode essential structural elements that characterize ABC transporters: two ATP binding domains and two substrate binding sites. Essential structural elements of ABCB4 normally contained within exon 12 and subsequent exons include both ATP binding sites and a substrate binding site.

A missense mutation in exon 15 of canine *ABCB*4 was identified in the one affected Shetland Sheepdog that did not harbor *ABCB*4 1583_1584G. This SNP results in a nonhomologous amino acid substitution (alanine to serine) in exon 15 which may affect tertiary protein structure. However, this mutation was also present in 9 of the 21 unaffected Shetland Sheepdogs and 10 of the 15 affected Shetland Sheepdogs, so its significance is unclear. No obvious differences were apparent in disease severity or biochemical parameters in the affected dogs with the mutation in exon 15.

#### Confirmation of Insertion by Allele Specific PCR

**Figure 3:**
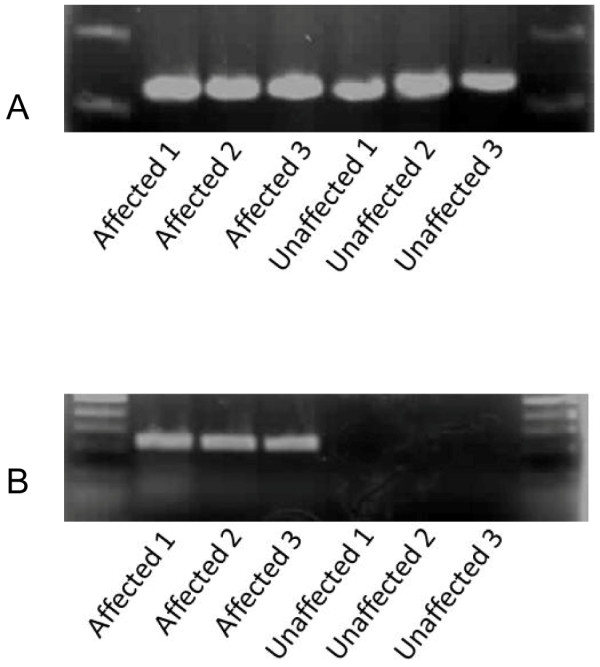
**Representative gels containing amplified DNA of canine *ABCB*4 from 3 affected (diagnosed with gallbladder mucocele) and 3 unaffected Shetland Sheepdogs**. Allele specific primers amplified both wildtype (A) and mutant (B) alleles in affected Shetland Sheepdogs, but only wildtype sequence was amplified in unaffected Shetland Sheepdogs.

To confirm the presence of *ABCB*4 1583_1584G as well as determine the genotype of each dog, allele specific primers were designed and used to amplify the region of interest in exon 12 (Figure 3). All dogs harboring the insertion were heterozygous at the mutant allele suggesting a dominant mode of inheritance with incomplete penetrance. None of the dogs in the study were homozygous for the mutant allele. Genotype frequencies are shown in Table 3.

**Table 3:** *ABCB*4 genotype frequencies in gallbladder mucocele affected and unaffected animals.

	Shetland Sheepdog (affected)	Shetland Sheepdog (unaffected)
*ABCB*4 1583_1584G (wildtype)	1	20
*ABCB*4 1583_1584G (heterozygous)	14	1
*ABCB*4 1583_1584G (homozygous)	0	0

	Other breeds (affected)	Other breeds (unaffected)
*ABCB*4 1583_1584G (wildtype)	0	20
*ABCB*4 1583_1584G (heterozygous)	3	0
*ABCB*4 1583_1584G (homozygous)	0	0

## Discussion

Over three dozen disease-causing mutations in human ABCB4 have been described [5,7,9,10]. The disease spectrum ranges from severe (debilitating diseases of young children that require liver transplantation) to mild. Disease severity often depends on the nature of the mutation. Milder disease occurs when the ABCB4 gene mutation reduces but does not eliminate transport activity of the protein. Similarly, milder forms of disease exist in patients that are heterozygous for mutations that eliminate transporter activity (*i.e*., truncations).

The canine *ABCB*4 insertion mutation reported here results in a truncation that eliminates more than 50% of the protein. This mutation was significantly associated with the diagnosis of gallbladder mucocele in Shetland Sheepdogs as well as other dog breeds. The etiology of gallbladder mucoceles in dogs is currently unknown, but extrahepatic bile duct obstruction is not a common component of the disease (as has been reported in people with gallbladder mucoceles) [18]. The results reported here provide evidence that dysfunction of *ABCB*4 is likely involved. Hepatocyte PC transport, and therefore bile PC content, in dogs that harbor *ABCB*4 1583_1584G would be decreased compared to wildtype dogs. Biliary epithelial lining cells would be subjected to bile salt-induced injury because of diminished ability to form mixed micelles [19]. A universal physiologic response of epithelial linings to injury is mucinous hyperplasia, a histopathologic finding frequently described in dogs diagnosed with gallbladder mucocele. Furthermore, exposure to bile salts has been shown to stimulate mucin secretion in cultured canine gallbladder epithelial cells [20]. Thus, gallbladder epithelium in dogs that harbor *ABCB*4 1583_1584G undergoes greater exposure to unneutralized bile salts than that of wildtype dogs, resulting in greater mucin secretion, mucinous hyperplasia, and eventually mucocele formation.

Because gallbladder mucoceles are a relatively new disease condition in dogs, a "gold standard" diagnosis has not yet been defined. Inclusion criteria used in previous publications consist of surgical or necropsy diagnosis (macroscopic appearance), ultrasonographic diagnosis, and/or histopathological diagnosis (microscopic appearance) [14,15,21]. Each of these criteria has limitations for diagnosing gallbladder mucoceles. A number of ultrasonographic findings have been associated with gallbladder mucocele, and there is sometimes disagreement among ultrasonographers as to what constitutes a gallbladder mucocele. Additional confusion is created by terminology such as "early" or "developing" gallbladder mucocole. Because of the gallbladder's universal physiological response to irritation (*e.g*., mucus secretion), some might argue that even a histopathological diagnosis of gallbladder mucocele may generate some speculation. It seems reasonable, therefore, to entertain the possibility that our study population ("affecteds") might contain false positives and that our control population ("unaffecteds") might contain false negatives despite the fact that currently acceptable criteria were used to identify these populations. However, the statistical difference between groups was so dramatic (based on current criteria) that statistical relevance would still hold even if some errors exist in the study or control population based on diagnostic criteria that may be defined in the future. The association of *ABCB*4 1583_1584G with gallbladder mucoceles in dogs represents an important advancement in our understanding of the disease.

A number of other potential etiologies have been suggested for gallbladder mucoceles in dogs. These include primary or secondary motility disorders of gallbladder motility, a secondary complication of dyslipidemias (Shetland Sheepdogs and Miniature Schnauzers) in particular, and primary disorders of mucus-secreting cells [13]. Recently, hyperadrenocorticism was reported to be significantly associated with the diagnosis of gallbladder mucocele in dogs [21]. Our findings do not rule out other potential etiologies, and it is certainly possible that *ABCB*4 1583_1584G could be one of many contributing factors to gallbladder mucoceles in dogs.

Many of the dogs from our study and other studies were severely affected at the time of diagnosis with some dogs dying of their disease despite surgical intervention [13,15]. Our discovery of the insertion mutation in canine *ABCB*4 allows early identification of dogs predisposed to gallbladder mucocele formation. This creates a number of beneficial applications for dogs. Genotyping of young dogs for *ABCB*4 1583_1584G would allow veterinarians to closely monitor for development of a gallbladder mucocele in affected dogs. Surgical intervention could be performed earlier in the disease process before disease-induced morbidity places the patient at higher risk for intra- and post-operative complications.

Another benefit of genotyping dogs for the *ABCB*4 1583_1584G is the possibility of medical or dietary management to prevent or at least delay the onset of mucocele formation. Currently, no medical treatment options have been systematically evaluated for managing dogs with gallbladder mucoceles primarily because information regarding the etiology of the disease has been lacking. However, ursodeoxycholic acid has been suggested [22]. Some human patients with ABCB4-associated biliary disease benefit from treatment with ursodeoxycholic acid, a relatively hydrophilic and much less cytotoxic bile acid than most endogenous bile salts [4]. Studies to determine bile composition in wildtype dogs and dogs with the *ABCB*4 1583_1584G mutation should be performed in order to further characterize the disease. One would expect affected dogs to have bile with lower phospholipid concentrations than wildtype dogs, and thus a greater proportion of simple micelles rather than mixed micelles. These studies would also be important to determine how useful affected dogs would be as a model for the various biliary diseases in people that result from similar ABCB4 mutations.

The authors speculate that occurrence of gallbladder mucoceles in dogs is inherited in a dominant fashion with incomplete penetrance, however further research is required to confirm the mode of inheritance. While it is possible that the one unaffected carrier of the *ABCB*4 1583_1584G insertion may develop biliary disease in the future, there was no evidence of disease at 9 years of age. No dogs in this study population were homozygous for the mutation. Because a more severe phenotype is observed in people homozygous for mutations resulting in elimination of ABCB4 protein function, one would speculate that the same would be true for dogs. In people with PFIC (type 3), the disease manifests during early childhood and is fatal without a liver transplant [4]. It is possible that homozygosity for the mutation results in death of affected dogs either during embryonic development or in early puppyhood.

In conclusion, the *ABCB*4 1583_1584G is strongly associated with the diagnosis of gallbladder mucocele in dogs. Results of this study provide the first spontaneous animal model for studying a number of potentially lethal or severely debilitating hepatobiliary diseases in people that are also associated with ABCB4 dysfunction. This canine model may be useful for studying potential medical and/or dietary treatments for ABCB4-associated hepatobiliary diseases in people.

## List of abbreviations

ABC: adenosine triphosphate-binding cassette; ABCB4: adenosine triphosphate-binding cassette, subfamily B, member 4; PC: Phosphatidylcholine, G: guanine.

## Competing interests

The authors declare that a patent application has been filed by Washington State University listing two of the authors as inventors (KLM, JDM).

## Authors' contributions

JDM performed experiments; JSM and KRS assisted in acquiring and interpreting data; SNW performed statistical analysis; KLM conceived and designed the research project. All authors made critical revision of the manuscript for important intellectual content. All authors read and approved the final manuscript.
